# Supplementary material for: Slumber in a cell: honeycomb used by honey bees for food, brood, heating… and sleeping
Source: PeerJ. 2020 Aug 5;8:e9583. doi: 10.7717/peerj.9583 (PMC7414769; doi:10.7717/peerj.9583)
Supplement: Supplemental Information 1 [file peerj-08-9583-s001.docx]

**SUPPLEMENTAL MOVIES**

Movie 1. Infrared-sensitive video of bee sleeping inside cell. Bee, center, is facing left with venter facing up. Note worker bees maintaining (cleaning or building) other cells.

Movie 2. Infrared-sensitive video of eating inside cell, with mouthparts extended and body less fully inserted in cell. Bee is facing left with dorsum facing up. Note worker bees maintaining (cleaning or building) other cells.

Movie 3. Infrared-sensitive video of heating inside cell. Bee, lower left, is facing left with venter facing observer (sideways).

Movie 4. Infrared-sensitive video of sleeping and heating inside cells. Sleeping bee, center, is facing left with dorsum facing up, and is to be compared with heating bee, at right, facing right with dorsum facing observer (sideways).

Movie 5. Infrared-sensitive video of worker bee inside cell. Gray box obscures cell innards, and small light gray rectangle marks bee of interest. This was one of 30 modified video clips used to test reliability of identifying inside-cell behavior from what is visible outside the cell. (Behavior? Answer, below.)

Movie 6. Infrared-sensitive video of worker bee inside cell. Gray box obscures cell innards, and small light gray rectangle marks bee of interest. This was one of 30 modified video clips used to test reliability of identifying inside-cell behavior from what is visible outside the cell. (Behavior? Answer, below.)

Movie 7. Infrared-sensitive video of worker bee inside cell. Gray box obscures cell innards, and small light gray rectangle marks bee of interest. This was one of 30 modified video clips used to test reliability of identifying inside-cell behavior from what is visible outside the cell. (Behavior? Answer, below.)

Movie 8. Infrared-sensitive video of worker bee inside cell. Gray box obscures cell innards, and small light gray rectangle marks bee of interest. This was one of 30 modified video clips used to test reliability of identifying inside-cell behavior from what is visible outside the cell. (Behavior? Answer, below.)

Movie 9. Thermal imaging video of heating bee inside cell. The thorax is relatively hot, the abdomen is continuously ventilating, but the bee is otherwise immobile. Video plays close to actual time (30 images per second).

Movie 10. Thermal imaging video featuring many acts of heating and cell maintenance (cleaning or building) inside cells. Video captured 1 image per second.

Movie 11. Thermal imaging video featuring many acts of heating and cell maintenance (cleaning or building) inside cells. Heaters’ thoracic temperatures fluctuate over time (e.g., bee featured in Figure 9c is from this video at 10 s after 07:58 h and 2 min 48 s later). Video captured 3 images per second.

Movie 12. Thermal imaging video of workers maintaining (cleaning or building) cells. Each bright spot is a relatively hot thorax, but of a bee maintaining, not heating, cells. This video plays close to actual time (30 images per second), allowing the viewer to observe a behavior closely for what it is.

Answers to Movies 5-8: sleeping, heating, eating, and maintaining cell, respectively
